# TRPV2 Calcium Channel Gene Expression and Outcomes in Gastric Cancer Patients: A Clinically Relevant Association

**DOI:** 10.3390/jcm8050662

**Published:** 2019-05-11

**Authors:** Pietro Zoppoli, Giovanni Calice, Simona Laurino, Vitalba Ruggieri, Francesco La Rocca, Giuseppe La Torre, Mario Ciuffi, Elena Amendola, Ferdinando De Vita, Angelica Petrillo, Giuliana Napolitano, Geppino Falco, Sabino Russi

**Affiliations:** 1 Laboratory of Preclinical and Translational Research, IRCCS—Referral Cancer Center of Basilicata (CROB), 85028 Rionero in Vulture (PZ), Italy; pietro.zoppoli@crob.it (P.Z.); giovanni.calice@crob.it (G.C.); simona.laurino@crob.it (S.L.); vitalba.ruggieri@crob.it (V.R.); francesco.larocca@crob.it (F.L.R.); giuseppe.latorre@crob.it (G.L.T.); mario.ciuffi@crob.it (M.C.); 2 Department of Biology, University of Naples Federico II, 80138 Naples, Italy; elena.amendola@unina.it (E.A.); giuliana.napolitano@unina.it (G.N.); 3 Division of Medical Oncology, Department of Precision Medicine, School of Medicine, University of Study of Campania “Luigi Vanvitelli”, 80138 Naples, Italy; ferdinando.devita@unicampania.it (F.D.V.); angelic.petrillo@gmail.com (A.P.); 4 Biogem, Istituto di Biologia e Genetica Molecolare, Via Camporeale, 83031 Ariano Irpino (AV), Italy

**Keywords:** bioinformatics analysis, calcium transport, gastric cancer, gene expression, prognosis

## Abstract

Gastric cancer (GC) is characterized by poor efficacy and the modest clinical impact of current therapies. Apoptosis evasion represents a causative factor for treatment failure in GC as in other cancers. Since intracellular calcium homeostasis regulation has been found to be associated with apoptosis resistance, the aberrant expression of intracellular calcium regulator genes (CaRGs) could have a prognostic value in GC patients. We analyzed the association of the expression levels of 98 CaRGs with prognosis by the log-rank test in a collection of 1524 GC samples from four gene expression profiling datasets. We also evaluated differential gene expression in comparison with normal stomach tissue, and then we crossed results with tissue microarrays from the Human Protein Atlas. Among the investigated CaRGs, patients with high levels of *TRPV2* expression were characterized by a shorter overall survival. *TRPV2* expression was found to increase according to tumor stage. Both mRNA and protein levels were significantly higher in tumor than normal stomach samples. *TRPV2* was also associated with poor prognosis in the Lauren’s intestinal type GC and in patients treated with adjuvant therapy. Overall, we highlighted the relevance of *TRPV2* not only as a prognostic biomarker but also as a potential therapeutic target to improve GC treatment efficacy.

## 1. Introduction

Gastric cancer (GC) is the fifth and third most common type of cancer worldwide for incidence and mortality, respectively. Incidence varies across different countries, and it is higher in developing and Asian areas [1]. The high mortality rates associated with GC are mainly ascribable to the poor efficacy of available treatments, especially in advanced disease, as well as the absence of early stage symptomatology and the lack of screening tests, which mainly contribute to late-stage diagnosis. Indeed, about 75% of patients present with an advanced or metastatic cancer [2], and have a five year overall survival (OS) rate of only 4% [3]. The treatment efficacy is low for the first-line setting and is based on platinum compounds (cisplatin/oxaliplatin), which activate apoptosis by DNA crosslinking, plus a fluoropyrimidine (5-FU, capecitabine, and S-1) that causes cell death by thymidine synthesis inhibition. These are combined with docetaxel, a microtubules depolymerization inhibitor, or trastuzumab, an anti-epidermal growth factor 2 (HER2) monoclonal antibody [3]. Indeed, their clinical impact remains modest, and gives an OS increase of only 1–3 months [4,5,6,7]. A similar scenario has been reported after the administration of targeted drugs against the vascular endothelial growth factor receptor 2 (VEGFR2), the monoclonal antibody receptor antagonist ramucirumab, and the tyrosine kinase inhibitor apatinib, as second- and third-line drug regimens, respectively [8,9]. The poor efficacy of treatments based on a single drug or a combination therapy is due to the marked inter- and intra-tumor histopathological and molecular heterogeneity [10]. Nowadays, the concept of precision medicine, in which the molecular signature of individual tumors can be used to select the most suitable therapeutic approach, has become the fulcrum of modern oncology [11]. On this basis, GC patients may obtain a major clinical benefit from the identification of molecular targets that play a pivotal role in cancer cell growth and survival, and that could contribute to the therapeutic management of patients. Molecular characterization of GC will considerably improve patients’ OS and/or progression-free survival (PFS).

By means of high-throughput-omics techniques (genomic, proteomic, and metabolomic approaches), significant advances have been made to unveil the molecular features of GC and, among a multitude of perspectives, define new possible molecular targets [12,13]. In this view, one of the most relevant cellular processes in cancer cells is represented by intracellular calcium (Ca^2+^) concentration homeostasis. Since calcium is the most abundant second messenger in humans, it plays a role in the regulation of several physiological cellular events that are commonly altered in cancer biology: Cell cycle progression, cell migration, and apoptosis [14,15]. The possible role of intracellular Ca^2+^ imbalance in neoplastic disease has been shown in extensively studied tumors [16,17,18]. As an example, the activity of the inositol trisphosphate receptor (IP3R) Ca^2+^ channels is prevented by the anti-apoptotic properties of Bcl-2, which diminishes Ca^2+^ flux from endoplasmic reticulum (ER) by blocking IP3Rs or decreasing Ca^2+^ levels in the ER lumen [19]. An additional pro-survival mechanism is the regulation of cytosolic Ca^2+^ concentration by Ca^2+^ excess leakage from cells. This was observed in breast cancer, where over-expression of plasma membrane calcium-transporting ATPase 2 (PMCA2) confers resistance to apoptosis and is associated with a poor outcome [20]. However, the alteration of Ca^2+^ channels and transporter genes in GC has not been extensively studied. Indeed, although several authors found the existence of correlations between Ca^2+^ channel gene expression and the prognosis of patients [21,22,23,24], among the different subtypes of Ca^2+^ channel genes, only a limited number of them have been investigated.

In this study, we focused on 98 Ca^2+^ regulator genes to assess whether their expression was associated with GC patient outcomes in terms of OS and/or PFS survival. To this end, we analyzed a large collection of GC patients’ gene expression profiling data through an integrated bioinformatic data-processing procedure. To identify possible subgroup-specific signatures capable of directing patients towards tailored therapeutic options, several clinicopathological parameters (e.g., tumor stage, histological classification, treatment history, etc.) were employed for patients’ stratification. Furthermore, we cross-checked results from computational analysis with data from immunohistochemical tissue microarrays from the Human Protein Atlas. We found a small number of Ca^2+^ regulator genes (CaRGs) whose gene expression was significantly associated with differences in OS, PFS, or both in a large cohort of GC patients. We also observed increasing expression levels of prognostic CaRGs according to tumor stage and a marked difference of their expression between normal and tumor tissue specimens.

Overall, our results suggest that Ca^2+^ regulation-related signatures could have a prognostic significance and could potentially represent innovative and effective therapeutic targets in GC [18].

## 2. Experimental Section

### 2.1. Selection of Ca^2+^ Regulator Genes

Overall, 431 candidate genes were retrieved by a Gene Ontology (GO) [25] search with the term “calcium ion transport” as “biological process”. This list was refined to 98 elements that considered only genes characterized by Ca^2+^ permeability, intrinsic Ca^2+^ channel/transporter activity, or ones considered as an essential Ca^2+^ channel assembly subunit, and whose expression data were present in each dataset. These genes were indicated as calcium regulator genes (CaRGs).

### 2.2. Collection of Gene Expression Datasets

A large collection of gastric cancer patients’ transcriptomic data was analyzed by integrating several gene expression microarray datasets. Firstly, we considered the Kaplan-Meier plotter (KMplotter) online database [26]. It integrates gene expression data from five datasets (GSE14210, GSE14459, GSE22377, GSE29272, and GSE51105) and provides a total of 593 samples with overall survival and 359 with progression-free survival data. Additional microarray data containing gene expression profiles from 248 and 300 samples (GSE15460 and GSE62254) were downloaded from Gene Expression Omnibus (GEO) [13,27].

We compared results from the integrated microarray dataset analysis with those from the RNA sequencing data in The Cancer Genome Atlas Stomach Adenocarcinoma (TCGA–STAD) dataset (383 samples with overall and 320 with progression-free survival annotations) [12]. Clinical characteristics considered in this study are reported in Table S1.

In all datasets, samples were divided into two cohorts on the basis of each CaRG expression. We considered low-expression samples to be those falling into the lower quartile (Q1) of gene expression value distribution, whereas those included in the upper quartile (Q4) were considered high-expression samples.

### 2.3. Evaluation of Overall and Progression-Free Survival

We evaluated overall and progression-free survival differences between the two expression cohorts on the whole patient cohort as well as on clinicopathological subgroups (present in at least two datasets), such as histological subtypes, TNM classification, tumor stage, and treatment experience. To quantify the potential clinical impact on patients, we also estimated the median or, if cohorts did not reach median survival, the restricted mean (rmean) of survival times for each cohort. The number of prognostic genes was reduced by cross-match validation, which considered as reliable only genes that were significant in at least two datasets and were characterized by the same type of association with prognosis.

### 2.4. Differential Gene Expression Analysis among Tumor Stages

The online tool Gene Expression Profiling Interactive Analysis (GEPIA) was used to investigate the differences in gene expression levels among tumor stages in the TCGA–STAD dataset [28]. Analysis was also performed in-house on the GSE62254 and GSE15460 datasets. Data were graphically reported as violin plots using the ggplot2 R package.

### 2.5. Differential Gene Expression Analysis

Expression levels of candidate genes in tumor samples from the TCGA–STAD dataset were compared with those in normal stomach mucosa specimens from the Genotype–Tissue Expression (GTEx) project [29]. Box plots of expression values as counts per million (CPM) were generated and the log_2_ fold change (log_2_Fc) was calculated for each candidate gene. Mean gene expression levels in the two tissue types were reported as CPM values.

### 2.6. Evaluation of Protein Expression in Tissue Sections

The large collection of immunohistochemistry (IHC)-based images in the Human Proteome Atlas database was used to assess the protein expression pattern of our candidate genes in GC samples compared with normal stomach ones [30].

### 2.7. Statistical Analysis

Overall and progression-free survival differences between the two gene expression cohorts were evaluated by the log-rank test, hazard ratio (HR), and 95% confidence interval (CI) using survival and survcomp R packages to identify protective (HR < 1) or risky (HR > 1) genes. Datasets included in KMplot as well as the GSE15460 dataset were MicroArray Suite 5 (MAS5) normalized using the affy bioconductor library [26]. The GSE62254 expression dataset was normalized by Robust Multi-array Average (RMA) [31]. Differential gene expression among tumor stages was evaluated by the one-way ANOVA test. The differentially expressed genes between tumor and normal samples, retrieved by TCGA and GTEx, were obtained by implementing a pipeline combining EDAseq [32], RUVg [33], and EdgeR [34] packages to perform normalization, batch removal, and differential expression analysis. Statistical significance was set at *p*-value < 0.05 for each analysis. All in-house statistical analyses were performed with R [35].

## 3. Results

### 3.1. Role of Ca^2+^ Regulator Genes on Gastric Cancer Prognosis

To evaluate the involvement of intracellular Ca^2+^ homeostasis on patient outcome, we focused our studies on genes that were characterized by: (a) Ca^2+^ channel/transporter activity, (b) Ca^2+^ permeability, and (c) Ca^2+^ channel assembly (Table S2). Subsequently, we individually evaluated the association between 98 CaRGs and GC patients’ OS and PFS data. 

To this end, we analyzed survival times in four different gene expression datasets (three with microarray and one with RNAseq data, altogether 1524 samples) independently to compare, for each gene, the difference in survival between the high and low transcription level groups (Figure S1). 

**Figure 1:**
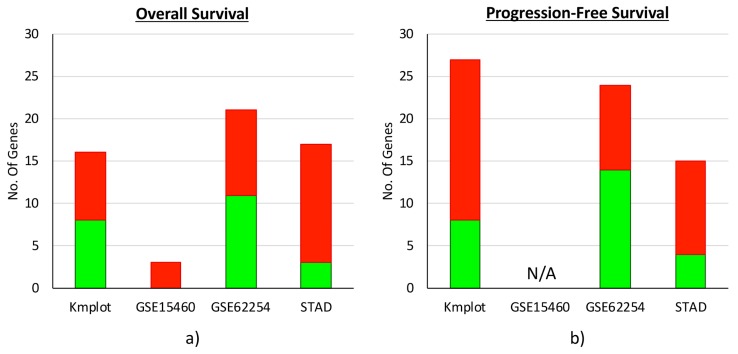
Ca^2+^ regulator genes with a prognostic significance in different datasets. Number of intracellular calcium regulator genes (CaRGs) that were associated positively (green) or negatively (red) with overall (**a**) and/or progression-free (**b**) survival differences between the high (Q4) and low (Q1) expression cohorts were reported for each dataset.

As shown in Figure 1a, we found 16 genes in the KMplot dataset (593 samples), 3 genes in the GSE15460 dataset (248 samples), 21 genes in the GSE62254 dataset (300 samples), and 17 genes in TCGA–STAD dataset (383 samples) whose high expression was associated with significant differences of GC patients’ OS. 

As regards to PFS (Figure 1b), we found 27 genes in the KMplot dataset (359 samples), 24 genes in the GSE62254 dataset (300 samples), and 15 genes in the TCGA–STAD dataset (320 samples) whose transcription levels were associated with survival differences. PFS could not be evaluated on the GSE15460 dataset because it was not reported.

Overall, across the datasets, we observed a slight prevalence of CaRGs whose high gene expression was associated with a poor outcome rather than a good prognosis.

### 3.2. Cross-Match Validation of Prognostic CaRGs

**Figure 2:**
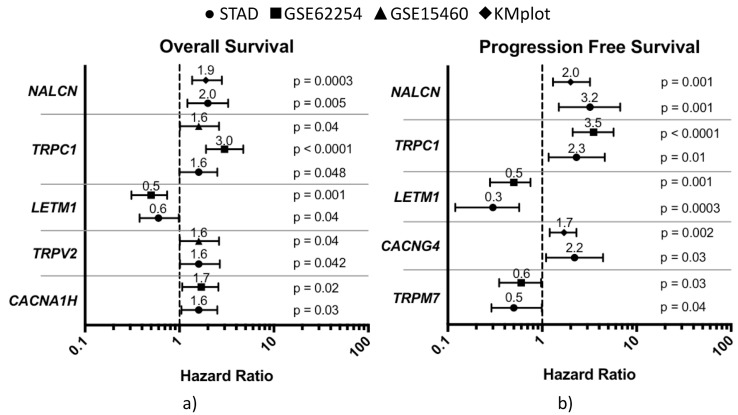
Forest plot of the risk associated with high calcium regulator gene expression considering all gastric cancer patients. Prognostic value, reported as hazard ratio, was shown only for genes that were significantly associated with overall survival (OS) (**a**) and/or progression-free survival (PFS) (**b**) differences in at least two datasets.

We mainly focused our study on genes that were significantly associated with survival differences in at least two GC datasets and that showed similar outcomes. As regards OS, a significant association between high expression levels and worse outcomes (HR > 1) was found for *NALCN*, *TRPC1*, *TRPV2*, and *CACNA1H* and, on the contrary, a better prognosis (HR < 1) was found to be associated with high expression of *LETM1* (Figure 2a).

Although there were some variations among datasets, the following genes were significantly associated with a shorter survival (differences expressed in months): *NALCN* (KMplot: ∆median −16.3; STAD: ∆median −43.5), *TRPC1* (GSE15460: ∆median −13.6; GSE62254: ∆rmean −32.9; STAD: ∆median −41.0), *TRPV2* (GSE15460: ∆median −19.1; STAD: ∆median −46.4), *CACNA1H* (GSE62254: ∆rmean −14.0; STAD: ∆median −43.5), and *LETM1* gene with longer survival (GSE62254: ∆median 23.2; STAD: ∆median 33.9) (Figure S2a).

As regards PFS, high expression levels of *NALCN*, *TRPC1* and *CACNG4* were significantly associated with shorter progression-free survival (loss of 7.1 to 36.4 months), whereas *LETM1* and *TRPM7* over-expression was associated with longer PFS (gain of 7.7 to 25.0 months) (Figure 2b). Specific results were as follows: *CACNG4* (KMplot: ∆median −7.1; STAD: ∆rmean −36.4), *NALCN* (KMplot: ∆median −14.6; STAD: ∆rmean −8.3), *TRPC1* (GSE62254: ∆rmean −35.7; STAD: ∆median −8.5), *LETM1* (GSE62254: rmean 25.0; STAD: rmean 16.8), and *TRPM7* (GSE62254: rmean 17.3; STAD: rmean 7.7) (Figure S2b). Finally, we found that only five genes passed the imposed constraint for OS or PFS. Interestingly, *NALCN*, *TRPC1*, and *LETM1* were associated with survival differences in both types of patient outcomes.

### 3.3. Impact of CaRGs on OS and PFS of Different Clinicopathological Subgroups of Patients

We also aimed to identify subgroup-specific signatures by evaluating the association between CaRGs and the outcome of GC patients classified according to different clinicopathological parameters (Table 1).

Only genes with a significant and concordant prognostic value in at least two datasets were considered reliable. Our analysis revealed that, in patients with intestinal type GC, *TRPV2* high expression samples were associated with a higher risk of death (OS reduction from 12.3 to 80.1 months). Patients with M0 stage tumors showed only *CACNA1H*, *SLC24A3* and *NALCN* associated with OS differences, while *ATP2A1*, *ATP13A2*, *TRPC1*, and *NALCN* proved to be significantly associated with OS in the N1–N3 tumor stage patient subgroup. In Stage IV GC patients, only *CACNA2D1* high expression was concordantly associated with a worse OS in at least two datasets. In patients treated with adjuvant chemotherapy, a slightly increased risk of death was observed in samples with high expression of *TRPV2* or *CACNB1*. Finally, we also considered patients who had undergone tumor resection without adjuvant chemotherapy. We found that patients overexpressing the *NALCN* gene were associated with a worse OS, while patients overexpressing *ATP2A1* and *LETM1* showed a better OS. Notably, the five genes previously described as significantly associated with both OS and PFS also recurred in one or more subgroups. On the other hand, we identified four genes peculiar of the different subgroups: *SLC24A3* for M0 stage, *ATP13A2* for N1+N2+N3 stages, *CACNA2D1* for stage IV, and *CACNB1* in patients who had undergone adjuvant chemotherapy.

As regards PFS (Table 2), in diffuse-type GC patients, we found that high expression of *P2RX1* and *LETM1* were associated with better PFS. *LETM1* high expression levels were associated with a reduced risk of disease progression in M0 patients, whereas high expression of *NALCN* and *TRPC1* showed association with a worse PFS. In the N1–N3 stage subgroup, high *TRPC1* expression was associated with worse PFS, while four genes (*CACNA1F*, *LETM1*, *CHRNA10*, and *P2RX1)* were associated with a reduced risk of disease progression. High *LETM1* levels were found to be associated with better PFS in both II and III tumor stage GC patients. We also found two other significant genes—*MCOLN2* and *TRPC1*—in tumor stage III patients. In patients treated with adjuvant chemotherapy, only *MCOLN2* resulted in being significantly associated with PFS. Finally, in resected GC patients not treated with any adjuvant chemotherapy, the high expression of *LETM1* and *GRIN3A* showed an association with better PFS in both datasets. Similarly to what was observed for OS, with the exception of *CACNG4* and *TRPM7*, genes that resulted in being significant in the whole cohort were also found to be relevant in one or more subgroups. Conversely, we found that *CACNA1F* and *CHRNA10* were significantly associated with PFS only in the N1–N3 subgroup, while *GRIN3A* significantly correlated with PFS in patients not treated with adjuvant therapy.

### 3.4. Differential Prognostic CaRG Expression among Tumor Stages

**Figure 3:**
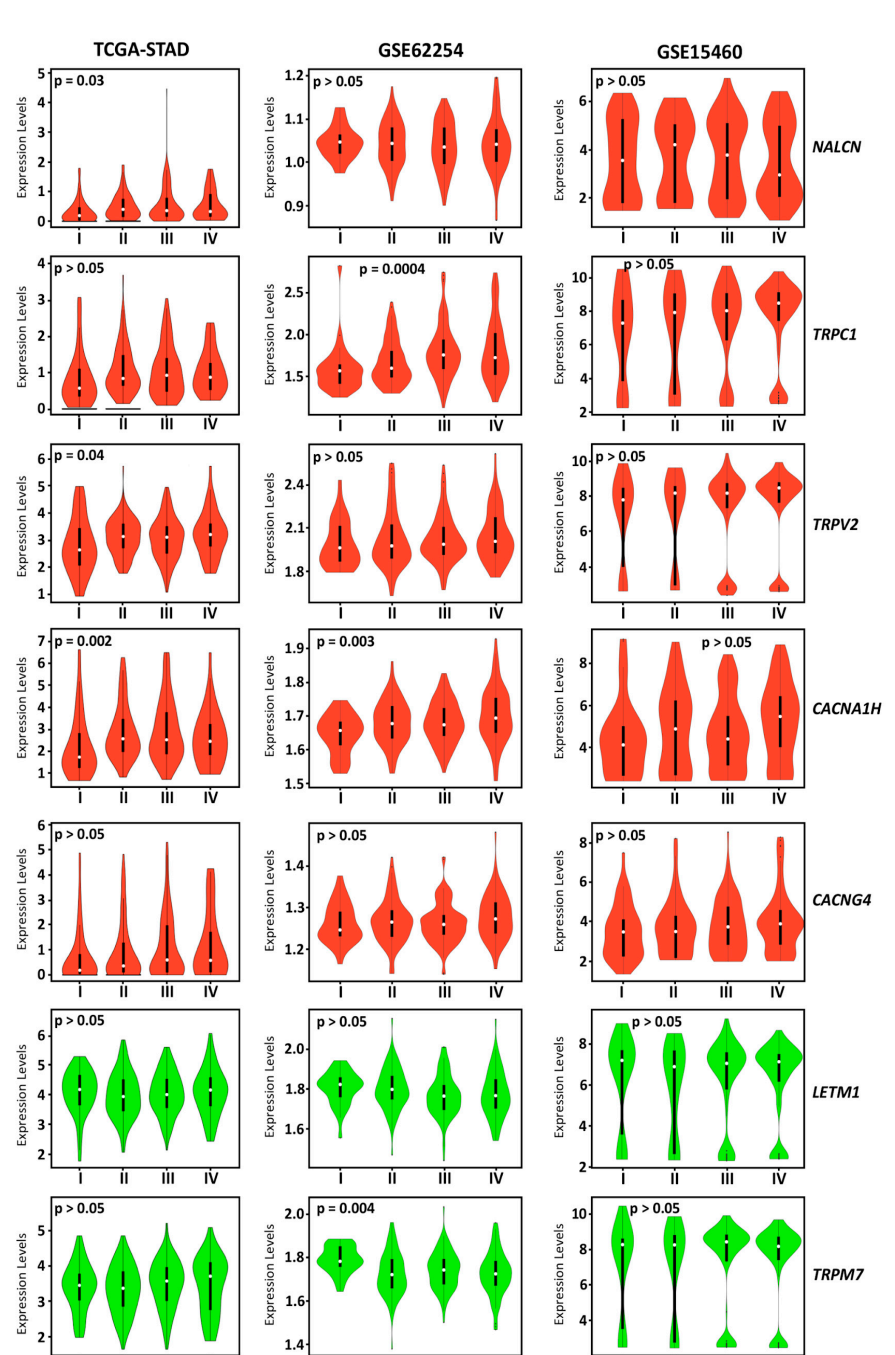
One-way ANOVA of prognostic CaRG expression across different tumor stages of gastric cancer in three different datasets (TCGA–STAD, GSE62254, and GSE15460). Red and green violin plots represent genes associated with poor and better prognosis in gastric cancer samples, respectively. Expression data are log_2_ transformed.

To identify potential tumor markers that could have a prognostic relevance on cancer spreading or that could be helpful in the treatment planning process, we further explored the association between the expression of all patients’ prognostic CaRGs and tumor stages in the GSE15460, GSE62254, and TCGA–STAD datasets (Figure 3). 

In accordance with their association with poor OS and/or PFS, significant differences were found for *NALCN*, *TRPV2*, and *CACNA1H* in the TCGA–STAD dataset. The expression of these genes was found to be correlated with tumor stage and showed progressively higher levels in more advanced tumor stages. On the other hand, no association with tumor stage was found for the remaining prognostic relevant genes. By analyzing the GSE62254 dataset, we found a significant association of prognostic CaRG expression with tumor stages for *TRPC1*, *CACNA1H*, and *TRPM7*, while no genes resulted in being significantly associated with tumor stages from the analysis of the GSE15460 dataset. Overall, our results indicated that high levels of *NALCN*, *TRPC1*, *TRPV2*, and *CACNA1H* could be suggestive of an advanced stage tumor. In the GSE62254 dataset, *TRPM7* showed a higher expression in tumor stage I, which is in agreement with its better prognosis association. 

### 3.5. Prognostic CaRGs in Tumor and Normal Stomach Mucosa Tissue Samples

**Figure 4:**
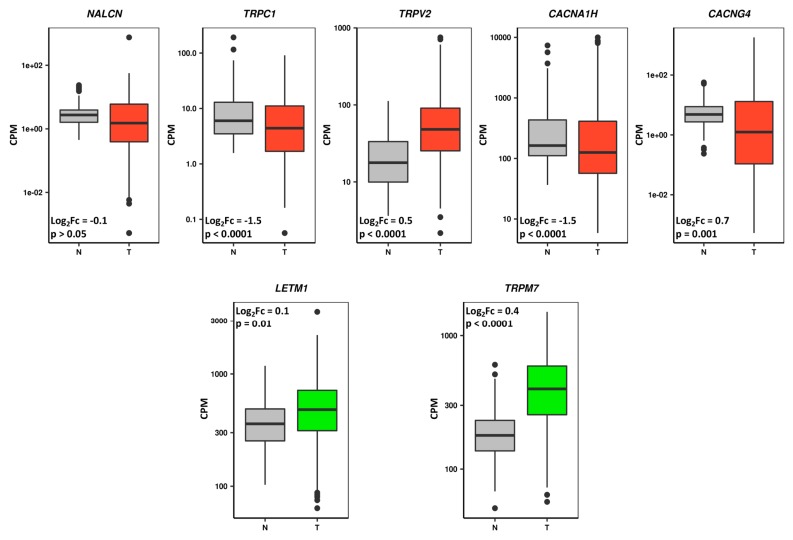
Differential prognostic CaRG expression between normal stomach mucosa (Genotype–Tissue Expression, GTEx, dataset) and gastric cancer (TCGA–STAD dataset) samples. Box-plots show gene expression levels as counts per million (CPM), and red and green boxes represent genes associated with poor and better prognosis of gastric cancer patients, respectively. Grey boxes indicate samples from normal subjects.

To verify whether the prognostic high expressed/poor survival CaRGs are up-regulated in GC samples versus normal gastric tissue specimens, we compared their expression levels in normal stomach mucosa samples from GTEx [29] with those reported in the TCGA–STAD dataset. At the same time, our analysis also allowed us to verify whether the high expressed/better survival CaRGs whose up-regulation correlates with a better prognosis are down-regulated in tumor versus normal samples (Figure 4). 

Among those genes, only *TRPV2* expression behavior was in line with the expected results. Although differentially expressed, *TRPC1*, *CACNA1H*, *CACNG4*, *LETM1*, and *TRPM7* expression behavior was not in line with their prognostic value. Finally, the *NALCN* gene showed no significant difference between GC and normal mucosa. 

Among the 10 genes that were significantly associated with prognosis and showed concordance among datasets in subgroup analyses, eight showed differential gene expression between GC and normal mucosa but only *SLC24A3*, *ATP2A1*, *CACNA1F*, *CHRNA10*, and *P2RX1* were in line with their prognostic value (Table 3).

As a result, one gene from the overall analysis and five genes from the subgroup analysis behaved coherently. The over-expression of these subgroup-specific CaRGs could be involved in the acquisition of a more aggressive tumor phenotype, and thus could be useful to tailor specific treatment for a particular cohort of GC patients.

### 3.6. Prognostic CaRG Protein Expression in Normal Stomach and Gastric Cancer IHC-Stained Tissue Sections

**Figure 5:**
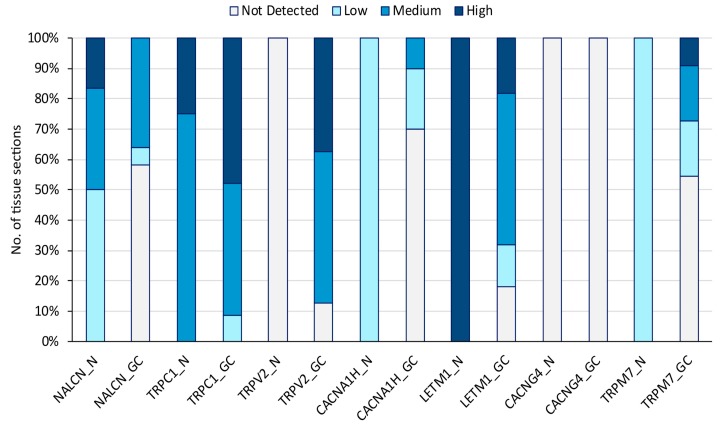
Differential protein expression of prognostic CaRGs in normal stomach (N) and gastric cancer (GC) tissue sections from the Human Protein Atlas (HPA) database. Colors represent the intensity of staining.

To assess whether, besides at mRNA levels, GC tissue was also characterized by high protein expression of the prognostic CaRGs, their protein expression levels were estimated in normal and gastric cancer tissue section images, retrieved respectively from the Tissue and Pathology Atlas of the Human Protein Atlas (HPA) antibody-based profiles database [30] (Figure 5). 

**Figure 6:**
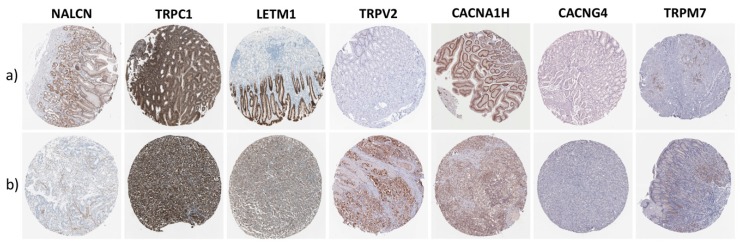
Representative immunohistochemistry (IHC)-stained tissue section showing the protein expression trend of prognostic CaRGs in (**a**) normal stomach and (**b**) gastric cancer tissue samples from the HPA database.

The most impressive findings were that the poor survival-associated gene *TRPV2* was not detected in glandular cells of normal gastric tissue sections, whereas its protein expression intensity in tumor samples ranged mostly from medium to high levels. Conversely, protein levels of the better prognosis gene *LETM1* showed an exactly opposite trend, since protein expression was identified as being lower (medium to absent) in 80% of cancer sections compared with normal tissue. Representative images of these findings are shown in Figure 6.

Among genes identified as significant in subgroup analysis, intriguing data emerged from the observation of stained tissue sections. ATP13A2 and MCOLN2 (Figure S3) protein expression levels were reduced in tumor samples when compared with normal ones, in line with the association of a better prognosis with a lower mRNA expression. Contrasting findings were observed for SLC24A3 and CACNB1, for which worse prognosis associated with high mRNA expression levels did not correspond to a high intensity protein staining in GC tissue sections. No differences in protein expression levels were found for ATP2A1, CACNA2D1, or CACNA1F, whereas no IHC sections were available for CHRNA10, P2RX1, or GRIN3A. 

## 4. Discussion

Currently, even though clinicopathological features such as age, sex, tumor type, margin status, and metastatic diagnosis provide useful parameters in clinical decision-making and in assessing cancer prognosis, they are not sufficient for a completely accurate outcome prediction in individual patients. In the era of precision medicine, research is oriented to unveil the molecular heterogeneity among patients and in the single tumor mass to dissect markers of cancer development, prognosis, and treatment susceptibility.

Due to its relevance as a second messenger and its role in critical cellular processes, Ca^2+^ has a pivotal role among the molecular players determining cancer fate. Thus, of particular interest is the study of Ca^2+^ permeable channels and transporters in cancer cells. Even though a growing number of studies have demonstrated the significant role of ion channels and transporters in gastric cancer, available data on the correlation between alterations of Ca^2+^ transport-related genes and patients’ prognosis in GC is still limited.

The availability of an increasing amount of gastric cancer global gene expression data allowed us to evaluate the association of the expression of 98 CaRGs with patient prognosis, comparing the prognostic value of each gene across different datasets to validate our results, by using stringent bioinformatic analyses. Overall, we analyzed gene expression data from 1524 samples of GC patients, and we found that among the 98 genes investigated, seven of them were significantly associated with prognosis. We also conducted a stratified analysis based on different clinicopathological parameters, evidencing, in some cases, discrepancies likely related to the relatively small number of patients included in the different subgroups, ethnicity, or inconsistency of clinical annotation among datasets. In addition, we characterized the expression of prognostic CaRGs both in GC samples at different stages and in normal stomach mucosa by using the TCGA–STAD and GTEx datasets, respectively. The same evaluation was performed on tissue microarray data from the HPA database.

We revealed that *NALCN* and *TRPC1* expression was associated with poor OS and PFS, whereas *LETM1* expression correlated with improved OS and PFS. We found that *TRPV2* and *CACNA1H* were associated with worse OS in two of four analyzed datasets. Conversely, *CACNG4* and *TRPM7* were significantly associated with poor and better PFS, respectively. Similarly, after cross-match validation, a prognostic value in distinct clinicopathological subgroups of GC patients (e.g., Lauren classification, TNM stages, and different treatment strategies) was observed only for a small number of the CaRGs that resulted in being significant in each dataset. Subsequently, we assumed that CaRGs whose expression levels were associated with different prognoses should be differentially expressed in tumor samples compared with normal stomach ones. To this end, we assessed candidate gene expression levels in the TCGA–STAD dataset compared with the GTEx dataset, and protein expression by comparing tumor and normal stomach tissue sections from the HPA database. After these evaluations, we found that only the *TRPV2* gene expression trend was coherent with our assumption as it was characterized by a negative prognostic value, increasing expression levels according to tumor stage, and higher expression levels of both mRNA and protein in tumor samples when compared with those from normal stomach mucosa. Since *TRPV2* expression correlated with prognosis in different subgroups of patients, in our opinion it is a reliable biomarker for GC molecular characterization.

The TRPV2 channel, a member of the transient receptor potential vanilloid (TRPV) subfamily of TRP channels, was found to be involved in tumor progression by a mechanism not yet completely clear. Physiologically, TRPV2 is highly permeable to Ca^2+^ and is activated by noxious heat above 52 °C, changes in osmolarity, and membrane stretch [36]. Its rectification current is dual, but the outward component, consisting of the efflux of cations from the cell, prevails [37]. TRPV2 activation induces its translocation from the endosome to the plasmatic membrane, inhibits cell proliferation, and induces necrosis and/or apoptosis, which can be impaired in the case of loss or alterations of TRPV2 signaling [38].

In accordance with our results, a negative impact on patient prognosis of *TRPV2* expression levels, and, in particular, a positive correlation with tumor stage, were reported in esophageal squamous cell carcinoma (ESCC) [39], hepatocellular carcinoma (HCC) [40], urothelial carcinoma (UC) [41], and prostate cancer (PC) [42]. Of the possible mechanisms, Monet et al. showed in vivo that TRPV2 activity promoted higher cytoplasmic Ca^2+^ concentration and cancer cell migration by induction of matrix metalloproteinases MMP2, MMP9, and cathepsin B in the development and progression of castration-resistant PC [42]. Caprodossi et al. suggested an additional mechanism by indicating the TRPV2/IGF-1/IGF-1R axis as the possible pathway capable of controlling UC growth and progression [41]. An interesting report showed that *TRPV2* is involved in the maintenance of cancer stem cells (CSCs) from ESCC and that tranilast, a *TRPV2*-specific inhibitor, decreased the population of CSCs, and is a possible candidate drug for combination therapy of ESCC [43]. In view of its outward rectification current and its over-expression in advanced stage cancer samples, it could be hypothesized that an alternative pathogenetic mechanism in which the up-regulation of this channel maintains intracellular Ca^2+^ at low concentration makes cancer cells more resistant to apoptosis [44]. Conversely, Nabissi et al. outlined an opposite picture in glioma. In particular, they observed a progressive reduction of both *TRPV2* mRNA and protein as tumor grade increased. Moreover, they found that *TRPV2* silencing was associated with ERK activation that drives glioma cell proliferation and apoptosis evasion by Fas and PI3K/Akt repression and Bcl-xL expression [36]. A possible explanation of this discrepancy may be related to the presence of TRPV2 variants that are able to interfere with the physiological functions of normal TRPV2 channels. This was reported for UC, in which a loss or reduction of the short TRPV2 variant was observed during cancer progression [41].

## 5. Conclusions

Our analyses suggest for the first time that the TRPV2 Ca^2+^ channel predicts the prognosis of gastric cancer patients, even in those with intestinal type GC. Since there is a need for innovative prognostic and predictive biomarkers in oncology, the preliminary evidence of an impact of *TRPV2* expression on patient survival should prompt further research on the biological mechanisms behind its function in GC. Our findings deserve further confirmation with in vitro and in vivo studies, and possibly large prospective cohorts of patients treated with TRPV2-targeted drugs. Although there are limitations of a retrospective investigation and a not homogeneous patient stratification among datasets, in our opinion, the constraints and the multiple validation steps performed in this work make this putative biomarker suitable for further investigations in this tumor type.

## Figures and Tables

**Table 1:** Association between CaRG expression and overall survival in different patient subgroups.

GENE	Study	Cases	Δmedian	HR	95% CI	Log-Rank P
**Intestinal Type**						
***TRPV2***	GSE15460	70	−80.1	1.9	1.0–3.7	0.04
	STAD	86	−12.3 *	2.1	1.1–5.4	0.04
***ATP2B3***	GSE62254	74	21.8	0.4	0.2–0.9	0.01
	STAD	86	−35.2	2.4	1.0–4.2	0.03
**Diffuse Type**						
***CACNB3***	KMplot	52	23.3	0.5	0.2–1.0	0.04
	STAD	38	−36.2 *	5.2	1.5–18.5	0.04
***GRIN2B***	GSE62254	72	24.5 *	0.5	0.2–0.9	0.02
	STAD	38	−31.5 *	4.1	1.4–11.3	0.004
***CACNA2D4***	GSE15460	44	44.2	0.4	0.2–0.9	0.03
	STAD	38	−13.3	2.9	1.0–8.8	0.046
**M Stage = 0**						
***CACNA1I***	GSE62254	136	20.3	0.5	0.3–0.8	0.003
	STAD	172	−44.1	1.7	1.0–2.9	0.048
***ATP2B3***	GSE62254	136	14.1	0.6	0.4–1.0	0.049
	STAD	172	−46.8	2.0	1.2–3.3	0.005
***CACNA1H***	GSE62254	136	−12.5	1.6	1.0–2.6	0.047
	STAD	152	−43.5	1.8	1.1–2.9	0.01
***SLC24A3***	GSE62254	136	−18.3	2.0	1.2–3.2	0.006
	STAD	172	−41.0	1.7	1.0–2.7	0.04
***NALCN***	KMplot	93	−29	2.3	1.4–4.0	0.001
	STAD	172	−43.3	2.1	1.2–3.6	0.006
**N Stage = 1+2+3**						
***ATP2A1***	GSE15460	69	67.5	0.4	0.2–0.8	0.008
	STAD	130	20.0	0.6	0.3–0.9	0.02
***ATP13A2***	GSE62254	130	38.7	0.5	0.3–0.8	0.007
	GSE15460	68	69.4	0.5	0.2–0.9	0.03
***TRPC1***	GSE62254	130	−30.9 *	2.7	1.7–4.3	<0.0001
	GSE15460	68	−13.3	1.9	1.0–3.6	0.04
***NALCN***	KMplot	89	−17.3	2.1	1.3–3.5	0.003
	STAD	130	−49.8	1.8	1.0–3.1	0.03
**Stage IV**						
***CACNA2D1***	GSE62254	38	−18.0	2.5	1.2–5.0	0.01
	STAD	20	−65.6	5.0	1.5–16.8	0.004
***SLC3A2***	GSE15460	36	−20.1	2.8	1.2–6.6	0.01
	STAD	20	19.9	0.2	0.1–0.7	0.005
**Treated with Adjuvant Chemotherapy**						
***TRPV2***	KMplot	76	−2.8	1.7	1–2.9	0.03
	STAD	14	−9.9 *	∞	1–∞	0.04
***CACNB1***	KMplot	78	−10.7	1.8	1.1–3	0.02
	STAD	14	−13.9 *	4.9	0.9–26.1	0.04
***TRPC1***	KMplot	76	5.3	0.5	0.3–0.8	0.008
	STAD	14	−11.8 *	7.4	0.8–67.6	0.04
***ITPR1***	KMplot	76	10.6	0.4	0.2–0.7	<0.0001
	STAD	14	−12.6 *	∞	1.8–∞	0.01
**Only Surgery**						
***ATP2A1***	GSE62254	110	54.2	0.6	0.3–1.0	0.04
	STAD	20	11.2	0.2	0.04–1.0	0.04
***LETM1***	GSE62254	110	63.8	0.4	0.3–0.7	<0.0001
	STAD	20	8.2	0.2	0.04–1.0	0.03
***NALCN***	KMplot	89	−15	2	1.1–3.4	0.008
	STAD	20	−13.9	4.9	1.0–24.0	0.03

Δmedian: difference in months between median survival time of high and low expression groups; *: rmean; HR: hazard ratio.

**Table 2:** Association between CaRG expression and progression-free survival in different patient subgroups.

GENE	Study	Cases	Δmedian	HR	95% CI	Log-Rank P
**Intestinal Type**						
***ATP13A3***	KMplot	63	−74.9	2.7	1.3–5.5	0.005
	STAD	66	17.3 *	0.2	0.04–1.0	0.03
***SLC24A4***	KMplot	66	71.1	0.5	0.2–0.9	0.02
	STAD	66	−39.3 *	10.4	1.3–82.7	0.006
**Diffuse Type**						
***P2RX1***	GSE62254	68	28.2 *	0.3	0.2–0.7	0.001
	STAD	32	31.3	0.3	0.1–1.0	0.04
***LETM1***	GSE62254	68	23.8 *	0.5	0.2–0.9	0.03
	STAD	32	11.4 *	0.1	0.02–1.1	0.03
**M Stage = 0**						
***LETM1***	GSE62254	128	27.6 *	0.4	0.2–0.7	0.0005
	STAD	146	13.3 *	0.3	0.1–0.7	0.002
***TRPC1***	GSE62254	128	−37.2 *	4.0	2.3–7.0	<0.0001
	STAD	146	−9.1	2.4	1.2–5.0	0.01
***NALCN***	KMplot	93	−23.4	2.3	1.4–3.8	0.001
	STAD	146	−8.8 *	3.3	1.5–7.3	0.001
**N Stage = 1+2+3**						
***CACNA1F***	GSE62254	122	20.2 *	0.5	0.3–0.9	0.01
	STAD	104	25.5	0.4	0.2–1.0	0.046
***LETM1***	GSE62254	122	27.2 *	0.4	0.3–0.7	0.002
	STAD	104	12.2 *	0.3	0.1–0.8	0.009
***CHRNA10***	GSE62254	122	16.6 *	0.6	0.4–1.0	0.04
	STAD	104	31.0 *	0.5	0.2–1.0	0.04
***TRPC1***	GSE62254	122	−32.9 *	3.0	1.8–5.0	<0.0001
	STAD	104	−40.4	2.4	1.1–5.4	0.02
***P2RX1***	GSE62254	122	16.2 *	0.6	0.3–0.9	0.03
	KMplot	88	10.9	0.5	0.3–0.9	0.02
***TRPV3***	KMplot	89	10.2	0.6	0.3–0.9	0.02
	STAD	104	−27.8 *	2.8	1.2–6.5	0.01
**Stage II**						
***LETM1***	GSE62254	44	24.1 *	0.3	0.1–1.0	0.04
	STAD	56	19.3 *	0.1	0.03–0.7	0.004
**Stage III**						
***MCOLN2***	GSE62254	46	35.4 *	0.3	0.1–0.7	0.005
	STAD	66	32.2 *	0.3	0.1–1.0	0.04
***LETM1***	GSE62254	46	26.1 *	0.3	0.1–0.8	0.008
	STAD	66	6.4	0.3	0.1–0.9	0.02
***TRPC1***	GSE62254	46	−38.3 *	4.0	1.7–9.6	0.0007
	STAD	66	−21.3	5.1	1.4–18.3	0.006
***ATP2B3***	GSE62254	46	24.2 *	0.4	0.2–1.0	0.04
	STAD	66	−10.8 *	3.1	1.1–8.9	0.03
**Treated with Adjuvant Chemotherapy**						
***MCOLN2***	GSE62254	36	26.5 *	0.1	0.01–0.9	0.01
	KMplot	17	n/a	0.2	0.03–0.9	0.02
***ORAI3***	GSE62254	36	−32.2 *	12.3	1.6–96.1	0.002
	KMplot	76	3.8	0.6	0.3–0.9	0.03
***TRPA1***	KMplot	78	−4.2	1.8	1.1–3.0	0.02
	STAD	14	15	0.2	0.05–0.9	0.02
***TRPC1***	KMplot	76	4.2	0.5	0.3–0.8	0.008
	STAD	14	−13.5	3.8	1.0–15.4	0.04
**Only Surgery**						
***RYR3***	GSE62254	100	−18.7 *	1.9	1.0–3.3	0.02
	STAD	22	7.2	0.4	0.1–1.0	0.048
***GRIN3A***	GSE62254	100	23.4 *	0.5	0.3–0.8	0.009
	STAD	22	28.5	0.3	0.1–1.0	0.03
***LETM1***	GSE62254	100	27.4 *	0.5	0.3–0.8	0.004
	STAD	22	12.4	0.2	0.08–0.8	0.009

Δmedian: difference in months between median survival time of high and low expression groups; *: rmean; HR: hazard ratio.

**Table 3:** Differential expression of subgroup-specific CaRGs between normal mucosa and GC tissue samples from GTEx and TCGA–STAD datasets, respectively.

GENE	Prognostic Value	Log_2_Fc	Mean CPM in Normal Samples	Mean CPM in Tumor Samples	*p* Value	FDR
*SLC24A3*	Negative	0.6	17.1	32.8	<0.0001	<0.0001
*CACNB1*	Negative	−0.3	15.1	15.9	0.0001	0.0002
*ATP2A1*	Positive	−0.7	2.0	1.0	<0.0001	<0.0001
*ATP13A2*	Positive	1.0	259.6	771.6	<0.0001	<0.0001
*P2RX1*	Positive	−1.6	22.5	3.6	<0.0001	<0.0001
*CACNA1F*	Positive	−1.0	1.5	0.4	<0.0001	<0.0001
*CHRNA10*	Positive	−1.1	1.2	0.5	<0.0001	<0.0001
*GRIN3A*	Positive	1.0	0.3	1.0	<0.0001	<0.0001

CPM: counts per million; Fc: fold change; FDR: false discovery rate.

## Supplementary Materials

The following material is available online at https://www.mdpi.com/2077-0383/8/5/662/s1, Table S1: Clinical characteristics of patients by gene expression dataset. Figure S1: Prognostic relevance of a representative CaRG mRNA expression in gastric cancer patients. Table S2: CaRGs list and selection criteria. Figure S2: Differences of OS (a) and PFS (b) duration between high and low expression cohorts of each prognostic CaRG. Figure S3: Differential protein expression in normal stomach and gastric cancer tissue sections from the HPA database of CaRGs with significant prognostic value in subgroup analysis.
