# Supplementary material for: TRPV2 Calcium Channel Gene Expression and Outcomes in Gastric Cancer Patients: A Clinically Relevant Association
Source: J Clin Med. 2019 May 11;8(5):662. doi: 10.3390/jcm8050662 (PMC6572141; doi:10.3390/jcm8050662)
Supplement: Supplementary file 1 [file jcm-08-00662-s001.pdf]

Journal of
Clinical Medicine

MDPI

Supplementary material

TRPV2 calcium channel gene expression and outcomes in gastric cancer patients: a clinically relevant correlation

Pietro Zoppoli [1], Giovanni Calice [1], Simona Laurino [1], Vitalba Ruggieri [1], Francesco La Rocca [1], Giuseppe La Torre [1], Mario Ciuffi [1], Elena Amendola [2], Ferdinando De Vita [3], Angelica Petrillo [3], Giuliana Napolitano [2], Geppino Falco [2,]* and Sabino Russi [1,]*

[1] Laboratory of Preclinical and Translational Research, IRCCS - Referral Cancer Center of Basilicata (CROB), Rionero in Vulture (PZ) , Italy; pietro.zoppoli@crob.it, giovanni.calice@crob.it, simona.laurino@crob.it, vitalba.ruggieri@crob.it, francesco.larocca@crob.it, giuseppe.latorre@crob.it, mario.ciuffi@crob.it, sabino.russi@crob.it

[2] Department of Biology, University of Naples Federico II, Naples, Italy; elena.amendola@unina.it, geppino.falco@unina.it, giuliana.napolitano@unina.it

[3] Division of Medical Oncology, Department of Precision Medicine, School of Medicine, University of Study of Campania "Luigi Vanvitelli", Naples, Italy; ferdinando.devita@unicampania.it, angelic.petrillo@gmail.com

* Correspondence: geppino.falco@unina.it; Tel.: +39 081 679092 (G.F.) and sabino.russi@crob.it; Tel.: +39 0972 726239 (S.R.)

Methods

Table S1. Clinical characteristics of patients by gene expression dataset.

Parameters	KMplot (n = 593)	GSE15460 (n = 248)	GSE62254 (n = 300)	STAD-TCGA (n = 385)
**GENDER**				
Female	138 (23.3)	87 (35.1)	101 (33.7)	133 (34.5)
Male	360 (60.7)	161 (64.9)	199 (66.3)	250 (64.9)
**AGE**				
(Mean ± SD)	-	65.4 ± 12.5	61.9 ± 11.3	65.4 ± 10.4
**TREATMENT**				
Only surgical	174 (29.3)	-	219 (73.0)	38 (9.9)
Adjuvant	153 (25.8)	-	80 (26.7)	27 (7.0)
**LAUREN'S HISTOLOGY**				
Intestinal	179 (30.2)	138 (55.6)	150 (50.0)	170 (44.1)
Diffuse	106 (17.9)	86 (34.7)	142 (47.3)	77 (20.0)
Mixed	25 (4.2)	22 (3.7)	8 (2.7)	-
**N STAGE**				
N0	38 (6.4)	-	38 (12.7)	116 (30.1)
N1+N2+N3	175 (29.5)	-	262 (87.3)	258 (67.0)
**M STAGE**				
M0	186 (31.4)	-	273 (91.0)	342 (88.8)
M1	31 (5.2)	-	27 (9.0)	25 (6.5)
**TUMOR STAGE**				
I	39 (6.6)	42 (16.9)	30 (10.0)	50 (13.0)
II	49 (8.3)	40 (16.1)	96 (32.0)	125 (32.5)
III	217 (36.6)	91 (36.7)	95 (31.7)	155 (40.3)
IV	74 (12.5)	73 (29.4)	77 (29.4)	39 (10.1)

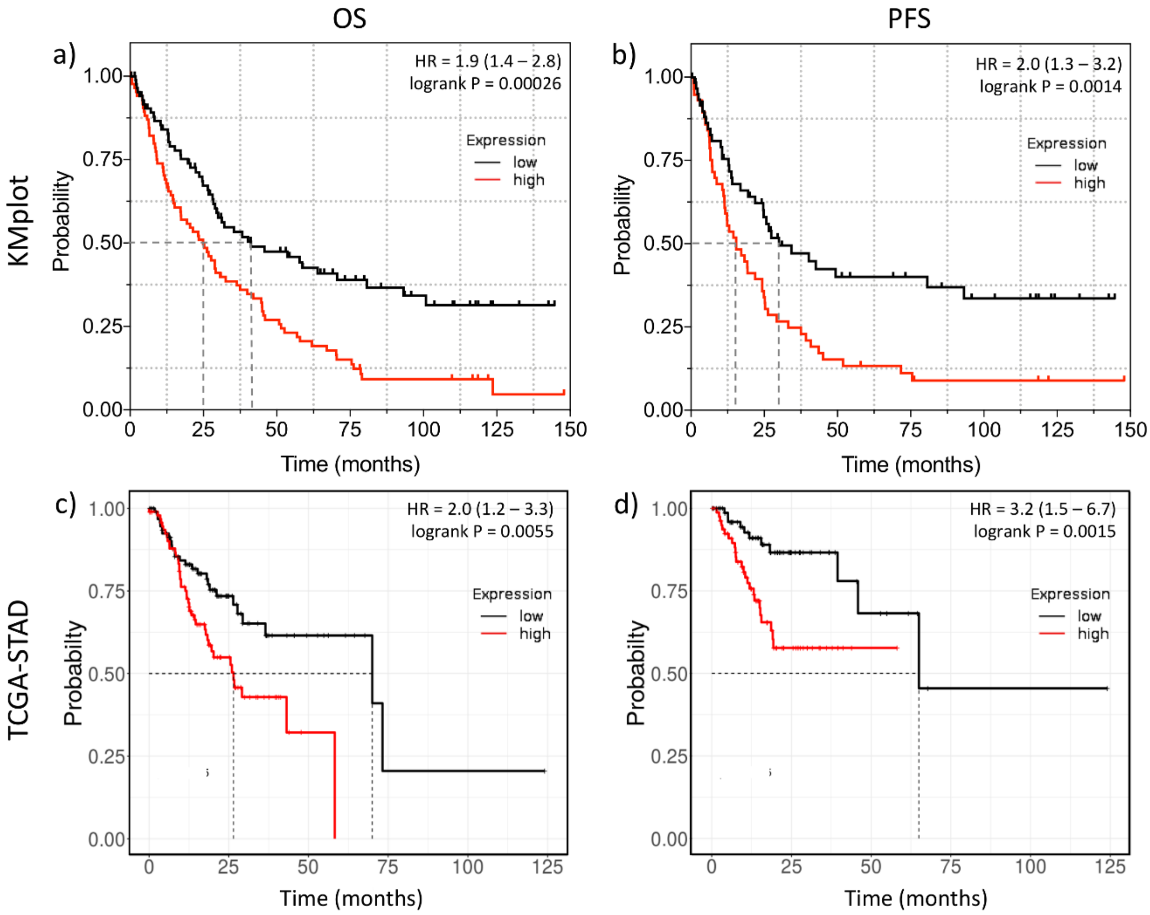

Figure S1. Prognostic relevance of a representative CaRG mRNA expression in gastric cancer patients. OS (**a**) and PFS (**b**) survival curves of the *NALCN* gene in the KMplot dataset (593 and 359 patients, respectively). OS (**c**) and PFS (**d**) survival curves of the *NALCN* gene in the TCGA–STAD dataset (383 and 320 patients, respectively). High and low expression cohorts represent samples falling in Q4 and Q1 of expression values.

Results

Table S2. CaRG list and selection criteria

Gene	Ca^{2+} channel/transporter	Ca^{2+} permeability	Ca^{2+} channels subunit
ANO6		x	
ATP13A2		x	
ATP13A3		x	
ATP13A4		x	
ATP13A5		x	
ATP2A1	x		
ATP2A2	x		
ATP2A3	x		
ATP2B1	x		
ATP2B2	x		
ATP2B3	x		
ATP2C1	x		
C19orf26			x
CACNA1B			x
CACNA1C			x
CACNA1D			x
CACNA1E			x
CACNA1F			x
CACNA1G			x
CACNA1H			x
CACNA1I			x
CACNA2D1			x
CACNA2D2			x
CACNA2D3			x
CACNA2D4			x
CACNB1			x
CACNB2			x
CACNB3			x
CACNB4			x
CACNG4			x
CACNG6			x
CACNG7			x
CACNG8			x
CCDC109B			x
CHRNA10		x	
GRIN1			x
GRIN2A			x
GRIN2B			x
GRIN2C			x
GRIN2D			x
GRIN3A			x
GRIN3B			x
ITPR1	x		
ITPR2	x		
ITPR3	x		
LETM1	x		
MCOLN2	x		
NALCN		x	
ORAI1			x
ORAI2			x
ORAI3			x
P2RX1			x

P2RX2			x
P2RX3			x
P2RX4			x
P2RX5			x
P2RX6			x
P2RX7			x
PKD1L1			x
PKDREJ	x		
RYR1	x		
RYR2	x		
RYR3	x		
SLC24A1	x		
SLC24A2	x		
SLC24A3	x		
SLC24A4	x		
SLC25A23	x		
SLC25A25	x		
SLC3A2	x		
SLC8A1	x		
SLC8A2	x		
SLC8A3	x		
STIM1			x
STIM2			x
TMC2	x		
TMCO1	x		
TMEM165	x		
TMEM37			x
TMEM63C		x	
TPCN1	x		
TPCN2	x		
TRDN			x
TRPA1		x	
TRPC1		x	
TRPC3		x	
TRPC4		x	
TRPC4AP			x
TRPC6	x		
TRPM2	x		
TRPM3	x		
TRPM6		x	
TRPM7		x	
TRPM8	x		
TRPV2		x	
TRPV3	x		
TRPV4		x	
TRPV6	x		

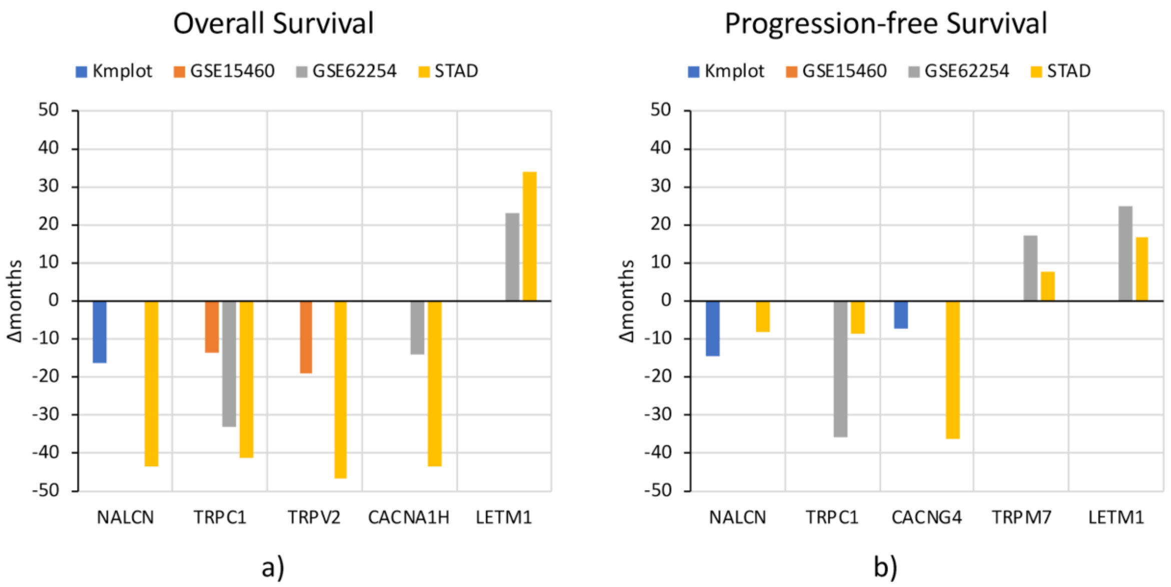

Figure S2. Differences of OS (**a**) and PFS (**b**) duration between high and low expression cohorts of each prognostic CaRG. Differences were calculated between median survival times of the two cohorts. If cohorts did not reach median survival, restricted mean (rmean) of survival times were considered.

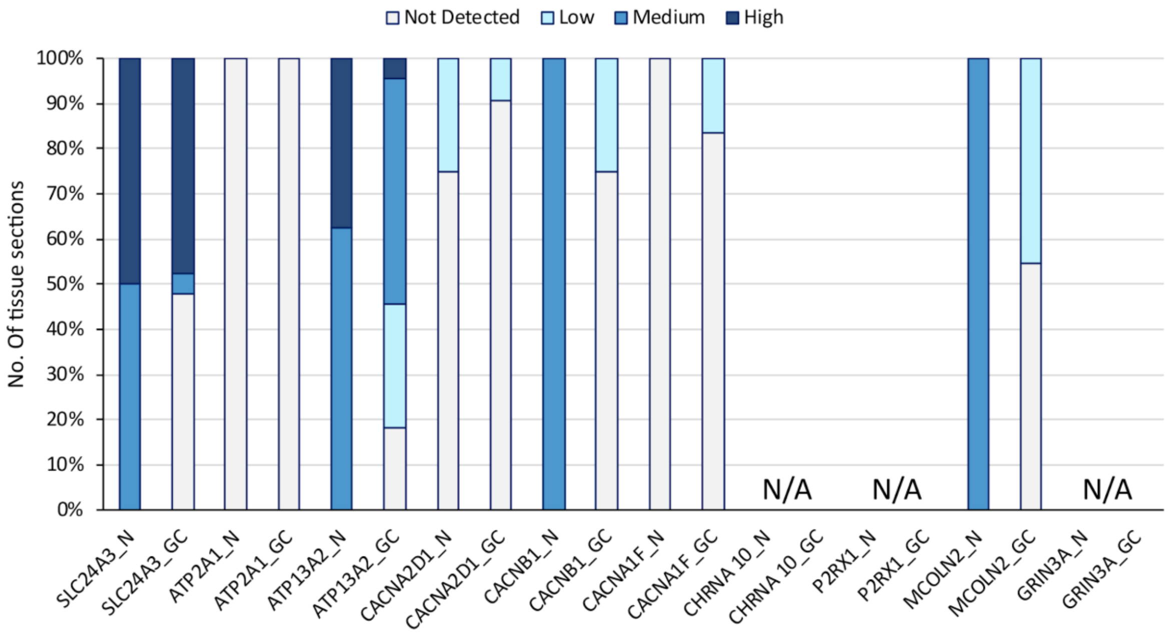

Figure S3. Differential protein expression in normal stomach (N) and gastric cancer (GC) tissue sections from the HPA database of CaRGs with significant prognostic value in subgroup analysis. Colors represents the intensity of staining.
